# Depressive symptoms, stigma and suicidal thoughts among people living with HIV/AIDS attending a Tertiary Hospital in Mwanza, Tanzania: A cross-sectional study

**DOI:** 10.1371/journal.pmen.0000311

**Published:** 2025-05-15

**Authors:** Matiko Mwita, Pendo Mkenda

**Affiliations:** 1 Catholic University of Health and Allied Sciences, Mwanza, TANZANIA; 2 Psychiatry department, Bugando Medical Centre (BMC), Mwanza, TANZANIA.; Universidad Técnica de Manabí: Universidad Tecnica de Manabi, ECUADOR

## Abstract

People living with HIV/AIDS (PLHIV) experience mental health challenges such as suicidal thoughts and depression. Moreover, PLHIV often face significant stigma, discrimination and social exclusions due to misconceptions surrounding the disease. This can leave them at risk for mental health problems, which may be associated with poorer treatment adherence and poorer overall health outcomes. A descriptive cross-sectional study was conducted in 300 PLHIV who attended the HIV clinic at Bugando Medical Centre (BMC). The Patient Health Questionnaire (PHQ-9) was used to assess depressive symptoms, and suicidal thoughts while stigma was assessed using a set of questions obtained from a working report measuring HIV stigma in Tanzania. Patients were recruited using a systematic sampling method between 1^st^ August 2021 and 31^st^ December 2021. The prevalence of depressive symptoms was 47.67%; and 37% had active suicide thoughts. Being male and aged 50 and above was associated with reduced odds of depressive symptoms, whereas being married and having a seropositive child was associated with increased odds of depressive symptoms. Being threatened with or experienced violent behaviors towards one’s self was significantly associated with suicidal thoughts. Among the participants 7.3% had not disclosed their HIV status, reason for non-disclosure was feelings of shame and guilt. Almost a half of the participants screened positive for depressive symptoms and more than one third reported suicidal thoughts. The high level of mental health difficulties in this population strongly supports the need for early screening to identify those who would benefit from appropriate interventions.

## Introduction

Depression is the most common neuropsychiatric complication associated with HIV/AIDS and people living with HIV (PLHIV) are more likely than the general population to develop depression [1]. The average prevalence of depression in the general population in Sub-Saharan Africa (SSA) is 5.5% [2]; however, it is higher in PLHIV [3,4]. Studies with large sample sizes and using internationally accepted diagnostic criteria for major depression in SSA report a prevalence rates of as low as 8% [5,6] to as high as 36% [7–9] among PLHIV. In Eastern Tanzania, a prevalence of 57.8% was found among women who initiated antiretroviral therapy (ART) [10].

Suicidal ideation or thoughts have also been reported as pervasive among individuals living with HIV/AIDS. Social stigma and socioeconomic burden have been reported as playing a major role in the development of suicidal thoughts [11]. Studies have found that 22.5% of PLHIV in Ethiopia reported suicidal ideation [12] and 45% of HIV positive men who have sex with men in India also reported persistent suicidal thoughts [13]. HIV/AIDS remains a highly stigmatized disease globally [14] with 64.3% of PLHIV reporting that they had experienced internalized stigma and 18.1% reporting that they had experienced external stigma [15].

Studies of PLHIV have reported that depression, suicidal thoughts and stigma are associated with failure to disclose HIV status, poor adherence to ART regimens, altered immune response, increased risk of HIV disease progression and mortality, mental health disorders, and overall poor health outcomes [16,17]. The aim of the present study was to investigate the prevalence of and factors associated with depressive symptoms, suicidal thoughts and stigma among PLHIV attending Bugando Medical Centre (BMC).

## Methodology

### Study design and settings

A cross-sectional descriptive study was conducted among PLHIV who attended the Care and Treatment Clinic (CTC) at BMC located in Mwanza, Northwestern Tanzania. BMC is the tertiary referral, teaching and research centre for the Lake and Western zones of the United Republic of Tanzania. It has more than 1000 beds with a catchment population of more than 15 million. The CTC serves about 1700 patients per month, providing primary HIV prevention, care and treatment services [18].

### Participants and recruitment methods

The study population (N = 300) were recruited from the BMC-CTC between 1^st^ August 2021 and 31^st^ December 2021. A systematic sampling method was used whereby every 2^nd^ client was approached. Trained research assistants approached potential participants, explained the aims of the study, and obtained their informed written consent to participate. Potential participants who were physically ill at the time of recruitment were not recruited. Those who obtained scores on our measure of depression that suggested a moderate or high risk of depression, or had active suicidal thoughts were referred to and/or escorted to the Psychiatry Clinic in BMC for further clinical evaluation and follow-up. Trained research assistants administered the research questionnaires to participants by reading all the questions and recording the participants’ responses. All of the participants who consented to participate in the study completed all of the study questionnaires and agreed to have their data included in the results.

### Research questionnaires

The questionnaires included a sociodemographic questionnaire (i.e., age, gender, place of residence (urban/rural), level of education, marital status, income, employment status and monthly income), the Patient Health Questionnaire-9 (PHQ-9) and a set of questions from a working report measuring HIV stigma in Tanzania published in 2005. The questions from this report were classified into three groups: stigma experienced from others, self-stigma and anticipated stigma. Each of the questions in the stigma experienced from others and self-stigma groups were examined independently to determine how many participants had endorsed the question by the sex of the participant. Anticipated stigma was examined by assessing whether the participant had disclosed their HIV status, and if they had not, the reasons for their non-disclosure [19].

The PHQ-9, which is a validated measure for screening for depression, was used to assess depressive symptoms. The questionnaire has nine questions related to depression. Scores range from 0 to 27 with higher scores indicating more depressive symptoms. Participants who scored less than 5 display minimal depressive symptoms. Scores between 5 and 9 are associated with mild depressive symptoms, whereas scores between 10 and 14 suggest moderate depressive symptoms. Scores between 15 and 19 indicate moderately severe depressive symptoms and scores between 20 and 27 represent severe depressive symptoms. The PHQ-9 has good internal consistency (Cronbach’s α = 0.78) [20] and has been extensively tested and widely used in various populations globally [21]. It has been used and tested in various African countries and translated into many languages including Swahili [20,22]. Suicidal thoughts were assessed using one question within the PHQ-9, which asks the client if they have ‘thoughts that you would be better off dead, or of hurting yourself’.

### Sample size and statistical analysis

To have 80% power to detect a doubling of prevalence in association with an exposure frequency of 25% [23] assuming an alpha value of 5%, a sample size of 300 participants was estimated using the Kish-Lisle formula of cross sectional studies [24]. Data entry was done using Epi Info software. Data was analyzed using Stata version 15 software for Mac. Categorical variables were summarized using frequencies and percentages, and continuous variables were summarized using means, SDs and ranges, or medians and IQRs. Descriptive analyses were conducted to examine the socio-demographic characteristics of the sample, and the prevalence and severity of depressive symptoms, stigma and suicidal thoughts. Logistic regressions with 95% CI were used to investigate the associations between sociodemographic factors (i.e., age, gender, and residency, level of education, marital status, income, employment status and monthly income) and depressive symptoms, stigma and suicidal thoughts, using standard cut-points on the symptom scales. Variables in the univariate analyses were considered for inclusion into the final multivariable logistic model if they were associated at a significance level of p < 0.2; the level of significance in the final model was set at p < 0.05. A model fit tests using Hosmer-Lemeshow for logistic regression was performed and found to be nonsignificant, indicating that the model was the best fit.

### Ethics

Ethical clearance for conducting this study was obtained from the Catholic University of Health and Allied Sciences Research and Ethics Committee, ethical clearance number 1949/2021. Permission to conduct the study was also obtained from BMC administration. Informed written consent was provided by all participants prior to their inclusion in the study.

## Results

### Sociodemographic characteristics

A total of 300 participants took part in the study. The mean age of the participants was 42.98 years (± 11.86 SD), with the majority 94 (31.3%) of the participants within the age group of 40–49 years. The majority of the participants were female, 215 (71.7%). Forty-four percent (n = 132) were married. Of those married, 84 (28.0%) had a spouse who was seropositive. Among the participants, 90 (30.0%) had lived with HIV/AIDS for less than five years since diagnosis, and 47 (15.7%) had children who were seropositive. Table 1 summarizes the sociodemographic characteristics of the study participants.

**Table 1:** Sociodemographic characteristics of PLHIV attending BMC-CTC.

Variable	Frequency (n)	Percentage (%)
**Sex**		
*Male*	85	28.3
*Female*	215	71.7
**Place of Residence**		
*Rural*	46	15.3
*Urban*	254	84.7
**Age groups**		
*18–29*	41	13.7
*30–39*	77	25.7
*40–49*	94	31.3
*50 and above*	88	29.3
**Marital status**		
*Never married*	168	56.0
*Married*	132	44.0
**Education level**		
*Never went to school*	27	9.0
*Primary education*	156	52.0
*Secondary education*	82	27.3
*College/University*	35	11.7
**Employment status**		
*Not employed*	72	16.3
*Employed*	71	23.6
*Self employed*	179	59.7
**Seropositive partner**		
*No*	216	72.0
*Yes*	84	28.0
**Seropositive child/children**		
*No*	253	84.3
*Yes*	47	15.7
**Years since diagnosis**		
*<5*	90	30.0
*5–10*	111	37.0
*11–20*	85	28.3
*>20*	14	4.7
**Years on ART**		
*< 5*	99	33.0
*5–10*	114	38.0
*11–20*	80	26.7
*>20*	7	2.30

### Prevalence and severity of depressive symptoms

Out of a possible maximum score of 27, participants had an average score on the PHQ-9 of 6.11 (SD 5.628, range = 0–23). Among the participants, 47.67% (n = 143) were experiencing symptoms of depression (PHQ-9 score above 4), while 52.33% (n = 157) displayed minimal symptoms of depression (PHQ-9 score of 4 and below); 24.33% (n = 73) of the participants had mild depressive symptoms (PHQ-9 score 5–9), 15.00% (n = 45) had moderate depressive symptoms (PHQ-9 score 10–14), 5.00% (n = 15) had moderately severe depressive symptoms (PHQ-9 score 15–19) and 3.33% (n = 10) had severe depressive symptoms (PHQ-9 score of 20 and above).

### Association between depressive symptoms and sociodemographic characteristics

In univariate analyses, gender, age, marital status, having a seropositive child, years since diagnosis and years on ART were significantly associated with depressive symptoms. Multivariable analysis (Table 2) revealed that male sex (AOR 0.8, 95% CI: 0.5, 0.8, p = 0.040), and being married (AOR 0.5, 95% CI: 0.2, 0.7, p = 0.046) significantly decreased the odds for depressive symptoms. Having a seropositive child was significantly associated with higher odds of depressive symptoms (AOR 1.8, 95% CI: 1.9, 3.3, p = 0.018). Those who have been diagnosed with the disease for 11–20 years were less likely to display depressive symptoms (AOR 0.2, 95% CI: 0.2, 0.9, p = 0.014). Model fit test using Hosmer-Lemeshow for logistic regression was performed and found to be the best fit with a p-value of 0.7096. Table 2 summarizes the association between depressive symptoms and sociodemographic characteristics of the study participants.

**Table 2:** Association between depressive symptoms and sociodemographic characteristics.

Variable	Depressive symptoms		Unadjusted OR (95% CI)		Adjusted OR (95% CI)	
***Sex***	**Yes (N %)**	**No (N %)**	**OR (95% CI)**	**P value**	**OR (95% CI)**	**P value**
Female	109(76.22)	106(67.52)	1.0		1.0	
Male	34(23.78)	51(32.48)	**0.6(0.4-0.7)**	**0.036**	**0.8(0.5-0.8)**	**0.040**
***Age groups (years)***						
18 – 29	25(17.48)	16(10.19)	1.0		1.0	
30-39	38(26.57)	39(24.84)	0.6(0.2-1.3)	0.229	0.9(0.4-2.0)	0.728
40-49	47(32.87)	47(29.94)	0.6(0.3-1.4)	0.241	0.9(0.3-2.1)	0.33
50 and above	33(23.08)	55(35.03)	**0.4(0.2-0.8)**	**0.014**	0.6(0.2-1.4)	0.218
***Marital status***						
Never Married	90(62.94)	78(49.68)	1.0		1.0	
Married	53(37.06)	79(50.32)	**0.6(0.4-0.9)**	**0.021**	**0.5(0.2-0.7)**	**0.046**
***Education level***						
Never went to school	17(11.89)	10(6.37)	1.0		1.0	
Primary education	74(51.75)	82(52.23)	0.5(0.2-1.2)	0.140	0.6(0.2-1.5)	0.271
Secondary education	39(27.27)	43(27.39)	0.5(0.2-1.3)	0.168	0.6(0.2-1.6)	0.288
College/University	13(9.09)	22(14.01)	**0.3(0.1-0.9)**	**0.046**	0.4(0.1-1.5)	0.189
***Employment***						
Never employed	39(27.27)	33(21.02)	1.0		1.0	
Employed	20(13.99)	29(18.47)	0.6(0.3-1.2)	0.151	0.6(0.3-1.6)	0.330
Self employed	84(58.74)	95(60.51)	0.7(0.7-1.9)	0.300	0.7(0.4-1.3)	0.220
***Seropositive partner***						
No	106(74.13)	110(70.06)	1.0		1.0	
Yes	37(25.87)	47(29.94)	0.9(0.5-1.4)	0.434	1.2(0.6-2.8)	0.634
***Seropositive child/children***						
No	115(80.42)	138(87.90)	1.0		1.0	
Yes	28(19.58)	19(12.10)	**1.8(1.9-3.3)**	**0.003**	**1.9(1.8-3.8)**	**0.018**
***Years since diagnosis***						
<5 years	57(63.33)	33(36.67)	1.0		1.0	
5–10	47(42.34)	64(57.66)	**0.4(0.2-0.7)**	**0.003**	1.4(0.3-6.9)	0.653
11–20	33(38.82)	52(61.18)	**0.4(0.2-0.7)**	**0.001**	**0.2(0.2-0.9)**	**0.014**
>20	6(42.86)	8(57.14)	0.4(0.1-1.4)	0.152	2.9(0.3-29.0)	0.361
***Years on ART***						
<5 years	63(63.64)	36(36.36)	1.0		1.0	
5–10	47(41.23)	67(58.77)	**0.4(0.2-0.7)**	**0.001**	0.3(0.1-1.4)	0.127
11–20	31(38.75)	49(61.25)	**0.3(0.2-0.7)**	**0.001**	0.3(0.1-1.7)	0.174
>20	2(28.57)	5(71.43)	0.2(0.0-1.2)	0.087	0.1(0.0-1.3)	0.078

### Prevalence and frequency of suicidal thoughts among study participants

**Fig 1:**
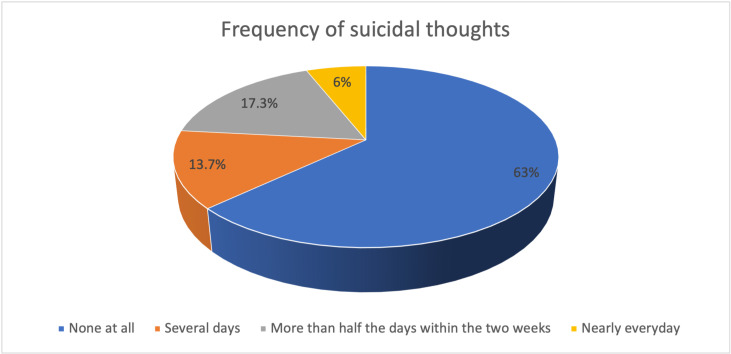
Frequency of suicidal thoughts among the study participants.

Results revealed that 37% (n = 111) of participants were having thoughts of suicide and self-injury, with 13.7% reporting suicidal thoughts several days in a week; 17.3% reported having thoughts of suicide on more than half of the days within the last two weeks, and 6.0% experienced suicidal thoughts nearly every day. Fig 1 summarizes frequency of suicidal thoughts among the study participants.

### Association between suicidal thoughts and sociodemographic characteristics

In univariate analyses, gender, age, marital status, education level, having a seropositive partner, having a seropositive child, years since diagnosis and years on ART were significantly associated with suicidal thoughts. Multivariable analysis revealed that male sex (AOR 0.3, 95% CI: 0.5, 0.7, p = 0.041), and being aged 50 years and above (AOR 0.5, 95% CI: 0.2, 0.6, p = 0.018) were associated with significantly decreased the odds of suicidal thoughts. Being married was associated with 1.3 times greater odds of suicidal thoughts (AOR 1.3, 95% CI: 1.4, 1.9, p = 0.031). Having a seropositive child was associated with 1.5 times greater odds of suicidal thoughts (AOR 1.5, 95% CI: 1.4, 2.1, p = 0.038). Those who have been diagnosed with the disease for more than 20 years (AOR 0.7, 95% CI: 0.1, 0.6, p = 0.016) and those on ART for more than 20 years (AOR 0.1, 95% CI: 0.0, 0.5, p = 0.021) were less likely to report suicidal thoughts. Model fit test using Hosmer-Lemeshow for logistic regression was performed and found to be the best fit with a p-value of 0.1542. Table 3 summarizes the association between suicidal thoughts and sociodemographic characteristics of the study participants.

**Table 3:** Association between suicidal thoughts and sociodemographic characteristics.

Variable	Suicidal thoughts		Unadjusted OR (95% CI)		Adjusted OR (95% CI)	
*Sex*	Yes (N %)	No (N %)	OR (95% CI)	P value	OR (95% CI)	P value
Female	87(78.4)	128(67.7)	1.0		1.0	
Male	24(21.6)	61(32.3)	**0.5(0.3-0.6)**	**0.027**	**0.3(0.5-0.7)**	**0.041**
***Age groups (years)***						
18 – 29	19(17.1)	22(11.6)	1.0		1.0	
30-39	35(31.5)	42(22.2)	0.7(0.1-1.9)	0.019	0.9(0.5-2.6)	0.619
40-49	18(16.3)	76(40.2)	0.7(0.4-1.3)	0.142	1.2(0.3-2.9)	0.342
50 and above	39(35.1)	49(26)	**0.6(0.3-0.9)**	**0.019**	**0.5(0.2-0.6)**	**0.018**
***Marital status***						
Never Married	72(64.9)	96(51)	1.0		1.0	
Married	39(35.1)	93(49)	**1.4(1.3-1.6)**	**0.016**	**1.3(1.4-1.9)**	**0.031**
***Education level***						
Never went to school	14(12.6)	13(6.9)	1.0		1.0	
Primary education	62(55.9)	94(49.7)	0.4(0.2-1.6)	0.341	0.7(0.4-1.8)	0.703
Secondary education	22(19.8)	60(31.8)	0.7(0.9-1.7)	0.601	0.2(0.1-1.3)	0.117
College/University	13(11.7)	22(11.6)	**0.1(0.1-0.6)**	**0.019**	0.2(0.1-1.7)	0.219
***Employment***						
Never employed	37(33.3)	35(18.5)	1.0		1.0	
Employed	11(9.9)	38(20.1)	0.7(0.1-1.9)	0.510	0.3(0.6-1.1)	0.130
Self employed	63(56.8)	116(61.4)	0.4(0.5-1.5)	0.316	0.9(0.3-1.6)	0.132
***Seropositive partner***						
No	86(77.5)	130(68.8)	1.0		1.0	
Yes	25(22.5)	59(31.2)	**1.4(1.2-1.6)**	**0.015**	1.1(0.2-1.9)	0.543
***Seropositive child/children***						
No	84(75.7)	169(89.4)	1.0		1.0	
Yes	27(2403)	20(10.6)	**1.4(1.2-1.9)**	**0.012**	**1.5(1.4-2.1)**	**0.038**
***Years since diagnosis***						
<5 years	21(18.9)	69(36.5)	1.0		1.0	
5–10	56(50.5)	55(29.1)	0.3(0.2-1.6)	0.163	1.5(0.2-4.1)	0.536
11–20	30(27.0)	55(29.1)	0.2(0.1-1.6)	0.051	0.3(0.9-1.6)	0.401
>20	4(3.6)	10(5.3)	**0.8(0.3-0.6)**	**0.012**	**0.7(0.1-0.6)**	**0.016**
***Years on ART***						
<5 years	48(43.2)	51(27.0)	1.0		1.0	
5–10	33(29.7)	81(42.9)	0.3(0.1-1.4)	0.104	0.2(0.4-1.3)	0.712
11–20	29(26.2)	51(27.0)	0.2(0.0-1.7)	0.301	0.3(0.4-1.9)	0.202
>20	1(0.9)	6(3.1)	**0.3(0.3-0.6)**	**0.010**	**0.1(0.0-0.5)**	**0.021**

### Association between depressive symptoms and suicidal thoughts among study participants

Depressive symptoms were associated with 2.7 increased odds of suicidal thoughts (OR 2.7, 95% CI: 2.1, 3.6, p < 0.001) (see Table 4).

**Table 4:** Association between depressive symptoms and suicidal thoughts.

Variable	Depressive symptoms		OR (95% CI)	
**Suicidal thoughts**	**Yes (N %)**	**No (N %)**	**OR (95% CI)**	**P value**
No	122(64.6)	67(35.4)	1.0	
Yes	97(87.4)	14(12.6)	**2.7(2.1,3.6)**	**<0.001**

### Stigma experienced by the study participants

Among the study participants, more women experienced each of the stigma experienced from others and self-stigma items compared to men. Approximately 40% (123/300) of women reported experience stigma from other, that is being gossiped about by people they knew. In addition, both men and women stated that they behaved a way to reduce the likelihood of people knowing their HIV status (125/300 for women, and 55/300 for men). More women (103/300) than men (45/300) admitted that their life goals changed after being diagnosed with HIV/AIDS. There was no significant association between sex of the participant and stigma experienced except for one item, which was being threatened with violence (p-value = 0.024). Women were significantly more likely to be threatened with violence than men. Table 5 summarizes the findings for stigma experienced from others and self-stigma in relation to sex of the participants.

**Table 5:** Stigma experienced from others and self-stigma in relation to sex of the participants.

Stigma Experienced from Others		Female	Male	Chi-square	P-value
*Abandoned by a spouse*	No	156	62	0.004	0.947
	Yes	59	23		
*Abandoned by family or sent away*	No	171	73	1.617	0.204
	Yes	44	12		
*No longer visited/visited less by family or friends*	No	164	65	0.001	0.972
	Yes	51	20		
*Isolated in your own household*	No	185	77	1.360	0.507
	Yes	30	8		
*Physically assaulted*	No	180	77	2.340	0.126
	Yes	35	8		
*Teased, insulted, or sworn at*	No	144	63	1.452	0.228
	Yes	71	22		
*Threatened with violence*	No	178	79	5.112	**0.024**
	Yes	37	6		
*Gossiped about by the people surrounding you*	No	92	45	2.530	0.112
	Yes	123	40		
**Self-Stigma**					
*Done things or behaved a certain way to prevent people from knowing HIV/AIDS status*	No	90	30	1.094	0.296
	Yes	125	55		
*Isolating oneself from family*	No	152	55	1.022	0.312
	Yes	63	30		
*Life goals or dreams changed since HIV/AIDS diagnosis*	No	112	40	0.618	0.432
	Yes	103	45		
*Negative feelings towards oneself*	No	122	46	0.171	0.680
	Yes	93	39		

### Self-stigma experienced by study participants

**Fig 2:**
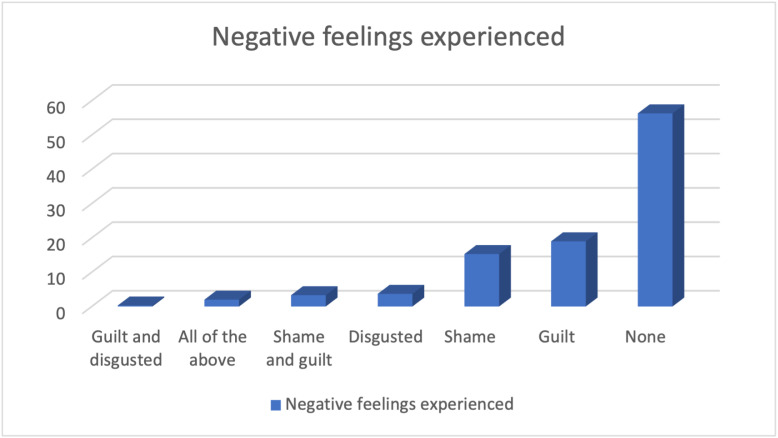
Negative feelings experienced by the study participants.

Forty-four percent of the participants had negative feelings towards themselves due to their HIV status and 19% reported feeling guilty due to their status. Fig 2 summarizes the negative feelings experienced by the study participants.

### Anticipated stigma among study participants

**Fig 3:**
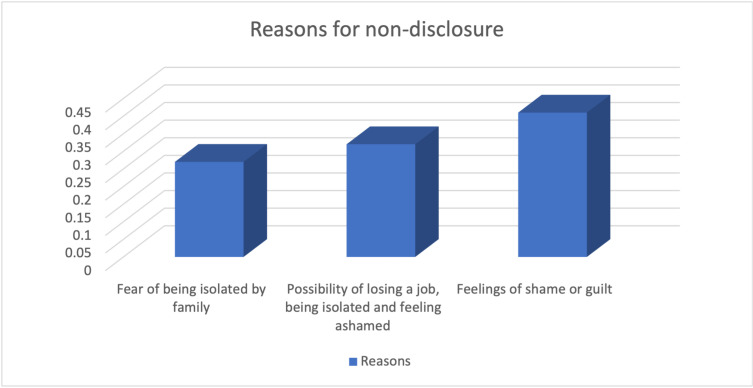
Reasons for non-disclosure of HIV status as reported by the study participants.

The results revealed that 7.3% of the participants had not disclosed their HIV status and most of those who had not disclosed their status indicated that their reasons for non-disclosure were feelings of shame and guilt (41%). Fig 3 summarizes the reasons for non-disclosure of HIV status as reported by the study participants.

## Discussion

The female to male ratio in this study was 2.5:1. The majority of participants were in the age group of 40–49 years, followed by those who were above 50 years of age. These findings are consistent with the Tanzania HIV indicator survey, which indicated that the prevalence of HIV was higher among women than men, with increased prevalence among adults peaking from 45-49 years in women and 30–49 for men [3]. Surprisingly, in this study more than a half of the participants, 56%, were not married. This is in contrast to a previous study done in rural Tanzania where majority of the participants were married [23].

The prevalence of depressive symptoms among people living with HIV (PLHIV) continues to be high as indicated by this study and previous research in the eastern part of Tanzania that reported a prevalence of 57.8% among women living with HIV/AIDS [10]. Lower prevalence rates have been reported in studies conducted in southwest Ethiopia (31%) [25], Sao Paulo, Brazil (42.3%) [26] and in Uganda (30.88%) [27]. The discrepancies among these studies could be due to differences in the tools used to assess depression, the study population, and the sample size.

Similar to previous research in Nigeria [28] and South Africa [29], we found that male sex was protective for developing depressive symptoms. Previous studies have revealed that sex was highly associated with depression; females were more likely to be depressed and this was associated with them being more prone to experiencing discrimination, being a single parent and poverty [25,26]. Being married has been reported to be a protective factor for developing depressive symptoms. This was observed in the present study and in other studies conducted in China [30] and East Africa [31]. Marriage has been associated with psychosocial supports and hence a decrease in the risk for depression.

This study observed that having a seropositive child was significantly associated with increased odds of depressive symptoms. This finding is similar to the findings observed in a study conducted in Brazil [26], which suggested that parents’ worry about their child’s outcome may increases the likelihood of them experiencing depression. It is notable that the present study revealed no significant association between depressive symptoms and having a seropositive spouse, which was reported as a risk factor in the a study conducted in Brazil [26].

We found that the prevalence of suicidal thoughts among PLHIV was 37%. This is similar to a prevalence of 39% that was found in a previous study conducted in Estonia [32], but is lower than the 45% reported in a study conducted in India [13] and higher than the prevalences of 15.1% found in Nigeria [33], 22.5% reported in Ethiopia [12], and 25% found in China [34]. The variability in the prevalence of suicide thoughts among the study populations in these different countries could be due to differences in the health and social supports system, and differences in ART adherence rates [35].

In this study being male was associated with reduced odds for depression, and also with reduced odds of suicidal thoughts. This has been observed in previous studies in Nigeria [28,36] and South Africa [29]. However, while being married was associated with reduced odds for depression, it was associated with increased odds for suicidal thoughts. This is in contrast to previous studies that revealed that being married was protective for both depression and suicidal thoughts [36] and being unmarried was strongly associated with the presence of suicidal thoughts [37]. The use of ART has been found to be associated with good HIV outcome and reduced psychiatry comorbidity [38,39]. This was consistent with our finding that those on ART for more than 20 years had lower odds of having suicidal thoughts, which was also reported in a study conducted in Ethiopia [40].

Stigma experienced by PLHIV remains a problem within communities as was evidenced in this study. In terms of self-stigma, most of the participants had revealed that they had disclosed their seropositive status; however, a small percentage, 7.3%, had not disclosed their HIV status as they were feeling shame and guilt about their status. The self-stigma reported by our participants was similar to what was observed in China, where participants reported internalized stigma due to their HIV status [15]. Similar findings have also been observed in Ethiopia [41] and other studies from LMICs [17].

This study revealed that, more females than males experienced each item related to stigma; however, there were no significant associations between the sex of the participant and the type of stigma experienced. Consistent with the findings of this study, a previous study conducted in Tanzania measuring stigma among PLHIV reported that more females were threatened with violence, abandoned by their spouse or family members and experienced verbal stigma compared to males [19].

### Strengths

We used valid and reliable questionnaires to collect data on symptoms of depression, suicidal thoughts, and stigma among PLHIV attending a tertiary hospital in Mwanza, Tanzania. We also examined several relevant predictors of symptoms of depression and suicidal thoughts.

### Limitations

The study was a cross-sectional in nature, thus the associations reported cannot be presumed to be causal. Moreover, the cross-sectional methodology relied on self-reports of symptoms. This could be associated with recall bias and under or over reporting of symptoms. Suicidal thoughts were assessed by participants’ responses to a questionnaire read to them by research assistants. Respondents may not have disclosed their actual thoughts about suicide due to the sensitivity of the question. Further, PHQ-9 is used for identifying depression symptoms rather than diagnosing or grading depression severity, future study involving a clinical diagnosis by a psychiatrist is recommended.

### Conclusion

The prevalence of depressive symptoms and suicidal thoughts was relatively high among PLHIV who attended the Care and Treatment Clinic at BMC with almost a half of the participants screening positive for depressive symptoms and more than one third reporting suicidal thoughts. Stigma was also high among the study population with females experiencing more stigma related behaviors. Depressive symptoms, suicidal thoughts and stigma among PLHIV can have significant health consequences, which emphasizes the need for early screening to identify those who would benefit from appropriate interventions.

## Supporting information

S1 Data Dataset used for statistical analysis of this manuscript. (DTA)
